# P-390. Burden of Surgical Site Infections with pathogens resistant to perioperative prophylaxis in orthopedic tumor surgery: Secondary analysis of the PARITY trial

**DOI:** 10.1093/ofid/ofae631.591

**Published:** 2025-01-29

**Authors:** Sabine Kuster, Michelle Ghert, Timothy O’Shea, Caleb Gottlich, Dominik Mertz

**Affiliations:** McMaster University and Hamilton Health Sciences, Binningen, Basel-Landschaft, Switzerland; McMaster University and the University of Maryland, Oakville, Ontario, Canada; McMaster University, Hamilton, Ontario, Canada; Texas Tech Uniiversity Health Sciences Center, Lubbock, Texas; McMaster University, Hamilton, Ontario, Canada, Hamilton, ON, Canada

## Abstract

**Background:**

Treatment of malignant bone tumors of the lower extremity often requires complex surgical reconstruction with the use of endoprostheses. These procedures are associated with an increased risk for surgical site infection (SSI). Here, we describe the risk factors for microbial resistance, characteristics and microbiology of SSIs in this population in a secondary analysis of the PARITY trial.

**Figure:**
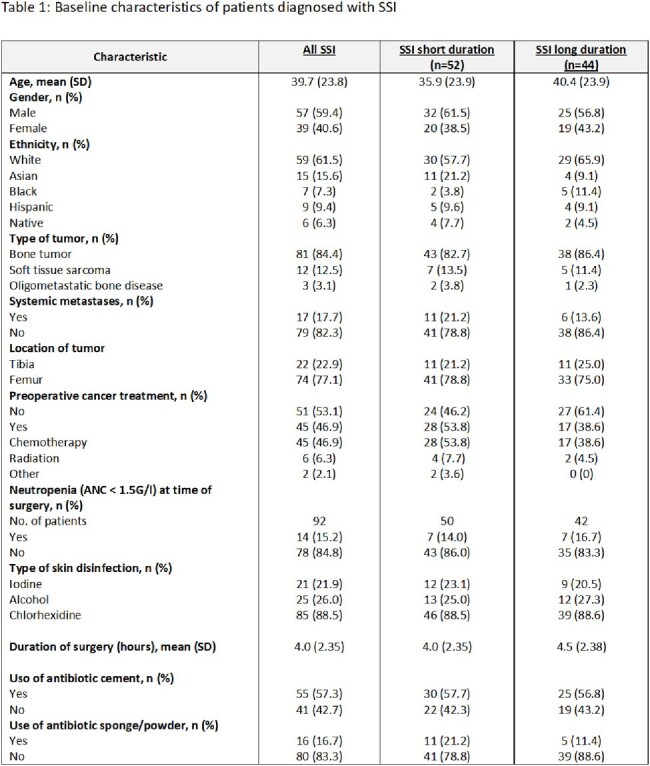

**Methods:**

The PARITY trial assessed the effect of short-term (24 hours) versus long-term (5 days) postoperative antibiotic prophylaxis with cefazoline or cefuroxime on SSI incidence in orthopedic oncology. Here, we evaluated the proportion of pathogens with presumed resistance to cephalosporine prophylaxis in this cohort. Resistance was defined either based on susceptibility testing, presence of intrinsic resistance mechanisms and/or based on the chosen antibiotics to treat a SSI episode.

**Figure:**
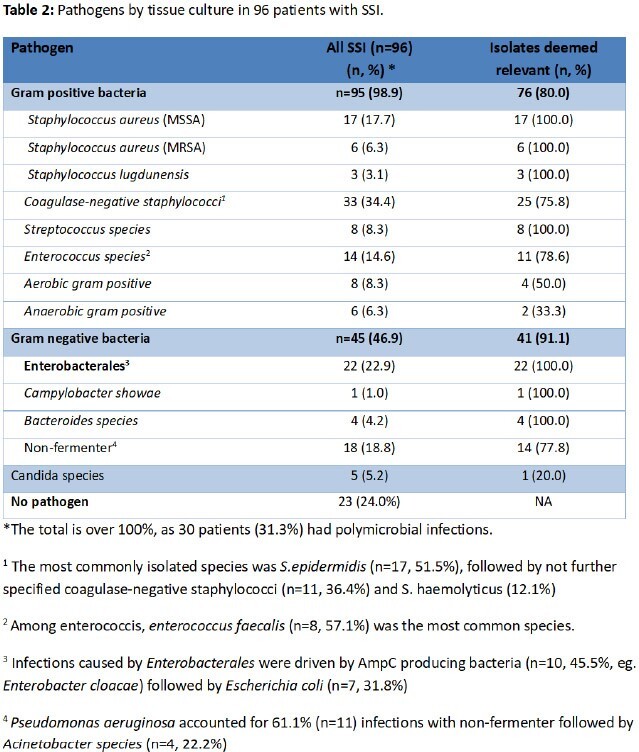

**Results:**

SSI were diagnosed in 96 of 604 patients (15.9%; Table 1). On average, SSIs occurred 77.4 days (IQR 81.25) after surgery. Among these, cultures were positive in 73 (76.0%). The most common pathogens were coagulase negative staphylococci (34.4%), followed by *S. aureus* (24.0%), and Enterobacterales (22.9%; Table 2). In 68.5% (n=50) SSI episodes, at least one pathogen was resistant to the prophylactic regimen. Polymicrobial infections were detected in 41.1% (n=30). Antibiotic cement was used in 40 (41.7%) patients, with resistance against the used antibiotic in 34 (85.0%). In the short-term group the proportion of resistant pathogens was lower (65.9% versus 71.9%; OR 0.75, CI 0.27, 2.06, p=0.385; Table 3). Proportionately more resistance was seen in patients with neutropenia (91.7% vs. 64.9%; OR 5.95, CI 0.72, 49.44, p=0.062), re-operation (80.0% vs. 66.7%; OR 2.00, CI 0.39, 10.27, p=0.328) and in those on postoperative antibiotics for more than 7 days prior to SSI diagnosis (75.8% vs. 62.5%; OR 1.88, CI 0.67, 5.21, p=0.169).

**Table 3:**
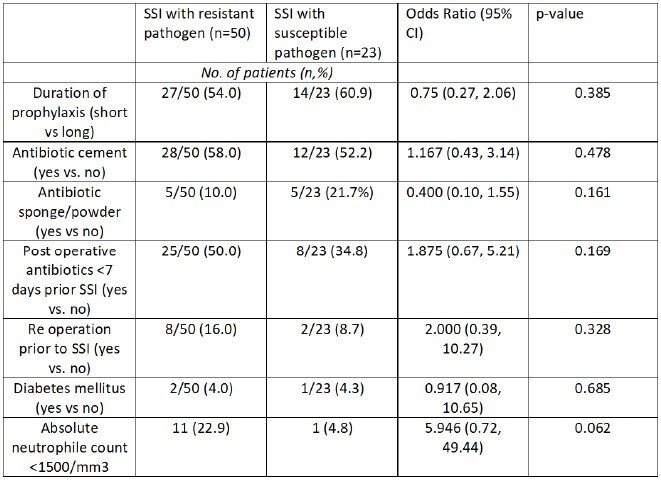
Risk factors for SSI caused by cephalosporin-resistant pathogens (univariate analysis)

**Conclusion:**

SSI due to pathogens resistant to the systemic or local prophylactic agents used are common in patients undergoing reconstruction for bone tumors. Several potential risk factors associated with resistance were evaluated, but the sample size limited our ability to draw final conclusions.

**Disclosures:**

**Sabine Kuster, MD**, Takeda Canada: Honoraria **Michelle Ghert, MD, FRCSC**, Stryker: Advisor/Consultant|Stryker: Grant/Research Support **Dominik Mertz, MD, MSc**, KCI Inc. USA: Grant/Research Support

